# Dynamics of Microbeams under Multi-Frequency Excitations

**DOI:** 10.3390/mi8020032

**Published:** 2017-01-24

**Authors:** Alwathiqbellah Ibrahim, Nizar Jaber, Akhil Chandran, Maloth Thirupathi, Mohammad Younis

**Affiliations:** 1 Mechanical Engineering Department, The State University of New York at Binghamton, 4400 Vestal Parkway E., Binghamton, NY 13902, USA; aibrahi4@binghamton.edu; 2 Physical Sciences and Engineering (PSE), King Abdullah University of Science and Technology, 23955-6900 Thuwal, Saudi Arabia; nizar.jaber@kaust.edu.sa (N.J.); thirupathi.maloth@kaust.edu.sa (M.T.); 3 Department of Aeronautical Engineering, Annasaheb Dange College of Engineering and Technology, Sangli, Maharashtra 416301, India; aac_aero@adcet.in

**Keywords:** multi-frequency, secondary resonances, superharmonic, electrostatic

## Abstract

This paper presents an investigation of the dynamics of microbeams under multiple harmonic electrostatic excitation frequencies. First, the response of a cantilever microbeam to two alternating current (AC) source excitation is examined. We show by simulations the response of the microbeam at primary resonance (near the fundamental natural frequency) and at secondary resonances (near half, superharmonic, and twice, subharmonic, the fundamental natural frequency). A multimode Galerkin method combined with the Euler-Bernoulli beam equation, accounting for the nonlinear electrostatic force, has been used to develop a reduced order model. The response of the cantilever microbeam to three AC source excitation is also investigated and shown as a promising technique to enhance the bandwidth of resonators. Finally, an experimental study of a clamped-clamped microbeam is conducted, demonstrating the multi-frequency excitation resonances using two, three, and four AC sources.

## 1. Introduction

The last decade has witnessed a growing research interest in down-conversion and the filtering of frequencies from radio frequency to intermediate frequency signals using microelectromechanical systems (MEMS) mixer-filters. Recent advancements in the field of micromachining technologies that yield high-*Q*, high frequency resonators and their applications as filters have shown the ability to realize the entire radio frequency RF-front end of a wireless transceiver in a single silicon chip. This has led to considerable interest in tuning and generating MEMS resonators of multiple resonance frequencies.

Several works have studied high *Q* and high resonance frequency resonators as filters and mixers [1,2,3,4,5,6,7]. Micro disk resonators have been shown to achieve *Q* > 2300 (at 193 MHz) [1], as well as *Q* > 2650 (at 1.156 GHz) [2]. Polysilicon ring resonators have been shown to reach frequencies up to 1.52 GHz with a *Q* > 2800 [3,4]. Mechanically coupled clamped-clamped microbeams have been introduced to enable MEMS mixer-filters, which convert a 200 MHz signal to 37 MHz [5]. Down-conversion and the filtering of frequencies up to 3.2 GHz has been demonstrated using a resonant mixer filter with potential for a single-chip receiver. Mixing through MEMS resonators have been demonstrated in [6,7].

MEMS mixer-filters exploit the nonlinearity of the electrostatic force with the drive voltage in the elelctromechanical resonators. Mixing and filtering functions are achieved simultaneously as the RF signals pass though the resonators. Parametric amplification has been used to improve the down conversion performance of a multimodal mixer-filter. Maximum amplification was reached for different modes due to the different values of the spring constant for each mode [8]. Resonators with capacitive mixer transducers were used to perform mixing and filtering of electrical input signals. Down conversion for RF signals from 40–200 MHz and 27 MHz were demonstrated in [5]. The method of multiple scales was used to calculate the response of a three degrees of freedom system under multi-frequency excitation [9]. An integrated complementary metal–oxide–semiconductor (CMOS) mixer was designed based on clamped-clamped beams and two different approaches were used to implement both up and down conversion mixers [10]. A coupled mode to analyze the acousto-optic diffraction with multiple waves at different carrier frequencies was developed in [11].

A methodology was introduced in [12] to provide analytical expressions for the mode shapes and natural frequencies of a coupled microbeam resonator filter. The response of a single-degree-of-freedom model under different types of nonlinearities was considered with the method of multiple scales for subharmonic resonance [13]. Softening and hardening behaviors were shown.

The dynamics behavior of a capacitive resonator under multi-frequency excitation was studied in [14]. Good agreement between the results of a single degree of freedom model and the experimental results was achieved. The ability to control the shift of the combinational frequency for any frequency range was also shown in [14]. The dynamics of clamped-clamped microbeams under two harmonic excitations were investigated analytically and experimentally in [15,16]. Higher amplitude and bandwidth were achieved near the main and higher order modes of vibrations. The dynamics of a torsional micromirror actuator was explored theoretically and experimentally under two-source excitations [17]. The results showed enhancement for the amplitude and bandwidth of the resonator near the primary resonance.

Research on the dynamics of microbeams has been investigated extensively in the literature over the past decade, for example in [18,19,20]. Higher pull-in voltage is achieved due to the intrinsic size dependence of the materials, while the natural frequency of the micro-plate is a function of its thickness [19,20]. Dynamics behaviors were investigated for different microbeams with coupling between in-plane, out-of-plane, longitudinal, rotational, transverse, and parametric vibrations [21,22,23]. The dynamics of an initially curved microbeam were investigated under electrostatic actuation [24]. These works have been conducted under single harmonic excitations.

It is noted that the dynamic behavior of cantilever microbeams under multi-source excitation has not been investigated before. Additionally, the use of multi-source excitation combined with secondary resonances has not been studied. Using more than two source of excitation has not been shown before.

In this paper, we explore the dynamics of a cantilever beam under multi-frequency excitation near primary and secondary resonances (subharmonic and superharmonic). We investigate the multi frequency excitation benefit in the down-conversion of a RF signal at a very high frequency of 960 MHz to a low frequency around 50 MHz. We also show experimental results for the response of clamped-clamped microbeams for multi-frequency excitations for cases of three and four AC sources.

## 2. Response to Two Harmonic Sources

### 2.1. Problem Formulation

**Figure 1:**
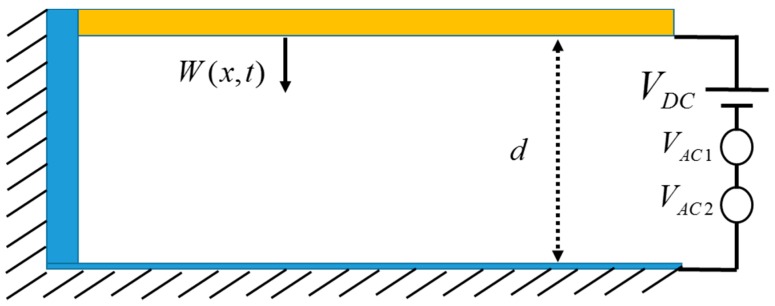
Schematic for the microbeam under direct current (DC) and two alternating current (AC) loads.

We consider a polysilicon microbeam, shown in Figure 1, which is clamped at one end and free at its other end. The beam is actuated by an electrode on the substrate at a gap width d from the lower electrode. The beam is modeled as an Euler-Bernoulli beam with length L, width b, and thickness h. The force function in the problem is modified by adding another harmonic AC load with a different excitation frequency. For a cantilever beam actuated with $V_{\mathrm{DC}}$ and two $V_{\mathrm{AC}}$ loads, the governing equation of motion is given by [25]

(1) $$\rho bh\ddot{\hat{W}}(\hat{x},\hat{t})+EI{\hat{W}}_{\hat{x}\hat{x}\hat{x}\hat{x}}(\hat{x},\hat{t})+\hat{c}\dot{\hat{W}}(\hat{x},\hat{t})=\frac{\varepsilon b{\left[V_{\mathrm{DC}}+V_{\mathrm{AC}1}cos({\hat{\Omega }}_{1}\hat{t})+V_{\mathrm{AC}2}cos({\hat{\Omega }}_{2}\hat{t})\right]}^{2}}{2{\left[d-\hat{W}(\hat{x},\hat{t})\right]}^{2}}$$

where ρ is the material density, I is the moment of inertia of the cross section, E is the Young’s modulus, $\hat{c}$ is a viscous damping coefficient, $V_{\mathrm{DC}}$ is the polarization voltage, $V_{\mathrm{AC}1}$ and $V_{\mathrm{AC}2}$ are the amplitudes of excitation of the first and second AC sources, respectively, ${\overset{\text{\textasciicircum{}}}{\Omega }}_{1}$ and ${\overset{\text{\textasciicircum{}}}{\Omega }}_{2}$ are their respective frequencies, respectively, and ε is the dielectric constant of the gap medium. In the equation, $\hat{W}\left(\hat{x},\hat{t}\right)$ is the beam displacement at location $\hat{x}$ and time $\hat{t}$, while the subscript letter indicates the spatial derivative and the dot indicates the time derivative. The associated boundary conditions are given by

(2) $$\hat{W}\left(0,\hat{t}\right)=0$$

(3) $${\hat{W}}_{\hat{x}}\left(0,\hat{t}\right)=0$$

(4) $${\hat{W}}_{\hat{x}\hat{x}}\left(l,\hat{t}\right)=0$$

(5) $${\hat{W}}_{\hat{x}\hat{x}\hat{x}}\left(l,\hat{t}\right)=0$$

For convenience, we introduce the following non-dimensional variables:

(6) $$W=\frac{\hat{W}}{d},\;x=\frac{\hat{x}}{l},\;t=\frac{t}{T},\;\Omega =\;\hat{\Omega }T,\;T=\sqrt{\frac{\rho bhl^{4}}{EI}}$$

Substituting Equation (6) into Equations (1)–(6) we end up with the following non-dimensional governing equation and boundary conditions:

(7) $$\ddot{W}(x,t)+W_{xxxxx}(x,t)+c\dot{W}(x,t)=\frac{\alpha_{2}{\left[V_{\mathrm{DC}}+V_{\mathrm{AC}1}cos(\Omega_{1}t)+V_{\mathrm{AC}2}cos(\Omega_{2}t)\right]}^{2}}{{\left(1-W(x,t)\right)}^{2}}$$

(8) W(0,t)=0

(9) $$W_{x}\left(0,t\right)=0$$

(10) $$W_{xx}\left(1,t\right)=0$$

(11) $$W_{xxx}\left(1,t\right)=0$$

where:

(12) $$c=\frac{12\hat{c}l^{4}}{ETbh^{3}}\;\alpha_{2}=\frac{6\varepsilon l^{4}}{Eh^{3}d^{3}}$$

Next, we expand the numerator of the electrostatic force as below

(13) $$F={\left(V_{\mathrm{DC}}+V_{\mathrm{AC}1}cos\left(\Omega_{1}t\right)+V_{\mathrm{AC}2}cos\left(\Omega_{2}t\right)\right)}^{2}\;=V_{\mathrm{DC}}^{2}+V_{\mathrm{AC}1}^{2}cos^{2}\left(\Omega_{1}t\right)+V_{\mathrm{AC}2}^{2}cos^{2}\left(\Omega_{2}t\right)+2V_{\mathrm{DC}}V_{\mathrm{AC}1}cos\left(\Omega_{1}t\right)+2V_{\mathrm{DC}}V_{\mathrm{AC}2}\cos\left(\Omega_{2}t\right)\;+V_{\mathrm{AC}1}V_{\mathrm{AC}2}\left[cos\left(\left(\Omega_{1}+\Omega_{2}\right)t\right)+cos\left(\left(\Omega_{1}-\Omega_{2}\right)t\right)\right]$$

The last term in Equation (13) contains the combinational terms: $\Omega_{1}+\Omega_{2}$ and $\Omega_{1}-\Omega_{2}$, which can lead to resonances similar to that of the main resonance when $\Omega_{1}+\Omega_{2}=\Omega_{n}$ or $\Omega_{1}-\Omega_{2}=\Omega_{n}$, where $\Omega_{n}$ is the resonance (natural) frequency. Such phenomena can be very useful in the fields of mixing and filtering, as explained in the introduction section.

Next, the Galerkin method [25] is applied to extract a Reduced Order Model (ROM). Thus, the beam deflection is expressed as

(14) $$W(x,t)=\sum_{1}^{n}u_{i}(t)\varphi_{i}(x)$$

where $u_{i}(t)$ is the modal coordinate and $\varphi_{i}(x)$ is the mode shape of the beam. The excitation source in the dynamic analysis is taken to be a combination of AC and DC loads. By substituting Equation (14) into Equation (7) and applying the procedure for the Galerkin method [25], we end up with the following Reduced Order Model equation

(15) $$\begin{array}{l} {\int_{0}^{1}\varphi_{j}\left(1-\sum_{1}^{n}u_{l}\varphi_{l}\right)}^{2}\left(\sum_{1}^{n}u_{i}\omega_{\mathrm{non},i}^{2}\varphi_{i}+\sum_{1}^{n}{\ddot{u}}_{i}\varphi_{i}\right)dx+c_{\mathrm{non}}{\int_{0}^{1}\varphi_{j}\left(1-\sum_{1}^{n}u_{l}\varphi_{l}\right)}^{2}\left(\sum_{1}^{n}{\dot{u}}_{i}\varphi_{i}\right)dx\\ =\alpha_{2}{\left[V_{\mathrm{DC}}+V_{\mathrm{AC}1}cos(\Omega_{1}t)+V_{\mathrm{AC}2}cos(\Omega_{2}t)\right]}^{2}\int_{0}^{1}\varphi_{j}dx \end{array}$$

A system of ordinary differential equations in the modal coordinate $u_{i}\left(t\right)$ can be extracted after executing the spatial integrals in Equation (15). To obtain good convergence in the dynamic response, we use at least three modes when simulating the dynamic response, which is done by integrating the ordinary differential equations with Long Time Integration (LTI). To explore the static and dynamics of the capacitive resonator, several case studies will be taken into consideration.

The static response problem can be achieved by setting all time derivatives and AC loads in Equations (7)–(11) to be equal to zero. This leads to the following:

(16) $$W_{sxxxx}\;\left(x\right)=\frac{\alpha_{2}{V_{\mathrm{DC}}}^{2}}{{\left(1-W_{s}\left(x\right)\right)}^{2}}$$

(17) $$W_{s}\left(0\right)=0$$

(18) $$W_{sx}\left(0\right)=0$$

(19) $$W_{sxx}\left(1\right)=0$$

(20) $$W_{sxxx}\left(1\right)=0$$

where, $W_{s}\left(x\right)$ represents the static response. Equations (16)–(20) are solved numerically, using the Galerkin procedure combined with a Newton Raphson method to obtain the static response.

### 2.2. Case I: Primary Resonance

The beam under study in this case will be assumed to have the geometric and physical parameters shown in Table 1. The beam is excited with two excitation sources: one is the RF signal, while the other is the Local Oscillator frequency (LO). Static and dynamics analysis in addition to the variation of the natural frequency of the beam will be investigated.

**Figure 2:**
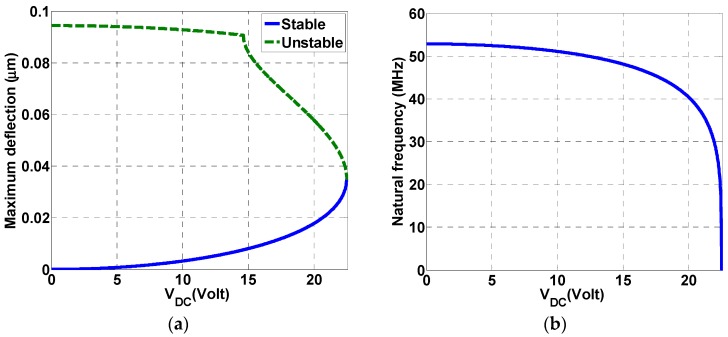
(**a**) Variation of the static deflection of the microbeam tip with the DC voltage; (**b**) Variation of the natural frequency of the microbeam with the DC voltage.

The variations of both the static deflection of the microbeam tip and the natural frequency with the DC voltage load are shown in Figure 2a,b, respectively. In Figure 2a, stable and unstable branches are shown for the static response for different DC loads until pull-in at a maximum static deflection of 35 nm is reached at a DC load of 22.5 V. Figure 2b shows the variation of the first natural frequency by varying the DC load. As seen from the figure, the natural frequency of the beam is near 52.85 MHz. This value is reduced as the applied DC load increases until reaching zero at pull-in around 22.5 V.

**Figure 3:**
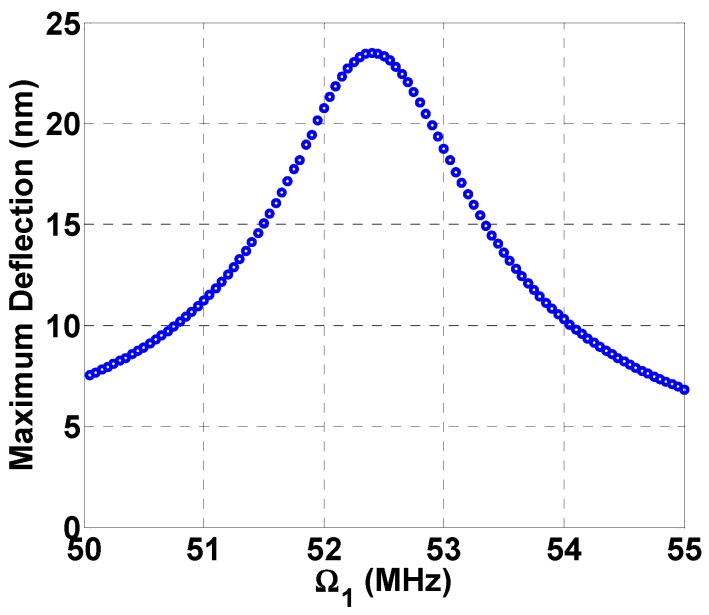
Frequency response curve near beam resonance, $V_{\mathrm{DC}}=5\;V$, $V_{\mathrm{AC}1}=2\;V$, $\;V_{\mathrm{AC}2}=0$, c=0.1.

To investigate the dynamic response of the cantilever resonator, the reduced-order model of Equation (16) is integrated numerically in time using three mode shapes for different DC and AC loads. First, the frequency response curve of the resonator is shown under a single frequency excitation, $V_{\mathrm{AC}2}=0$, Figure 3.

**Figure 4:**
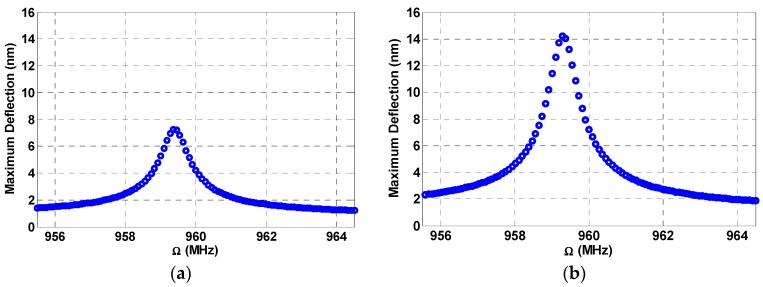
Multi frequency excitation (**a**) Lower amplitude: $V_{\mathrm{DC}}=2.5\;V$, $V_{\mathrm{AC}1}=0.5\;V$, $\Omega_{2}=906\;\mathrm{MHz}$, $V_{\mathrm{AC}2}=6\;V$, c=0.05; (**b**) Higher amplitude: $V_{\mathrm{DC}}=3.5\;V$, $V_{\mathrm{AC}1}=1\;V$, $V_{\mathrm{AC}2}=6\;V$, $\Omega_{2}=906\;\mathrm{MHz}$, c=0.05.

In the following dynamic analysis, the swept frequency is taken to be $\Omega_{1}$, while $\Omega_{2}$ is kept as the fixed one. Next, the cantilever beam is subjected to a DC load and two source AC loads. Figure 4 demonstrates down conversion where the combination resonances of the beam appear at much higher values compared to the primary resonance of Figure 3. Note that the fixed frequency is chosen such that when it is subtracted from the excitation frequency (near 960 MHz) it results in the main resonance frequency (near 52 MHz, as in Figure 3). The beam in this case oscillates at its resonance producing a frequency response at 52 MHz, thereby achieving the down conversion from the incoming input frequency of 960 MHz.

### 2.3. Case II: Secondary Resonances

In this section, we investigate the multi-frequency excitations near secondary resonance (superharmonic and subharmonic) for a cantilever beam actuated with electrostatic force [26]. The following geometric and physical parameters shown in Table 2 are used for the case study.

**Figure 5:**
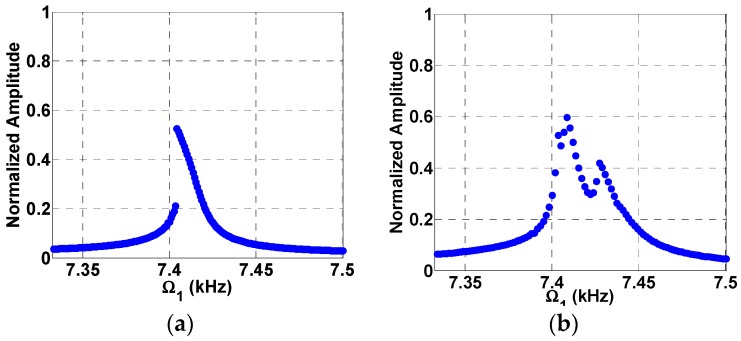
Frequency response curves near superharmonic resonances for $V_{\mathrm{DC}}=1\;V$, $V_{\mathrm{AC}1}=0.35\;V$ (**a**) Single source excitation at $V_{\mathrm{AC}2}=0$; (**b**) two-source excitation at $V_{\mathrm{AC}2}=0.1\;V$, $\Omega_{2}=7.5\;\mathrm{kHz}$.

**Figure 6:**
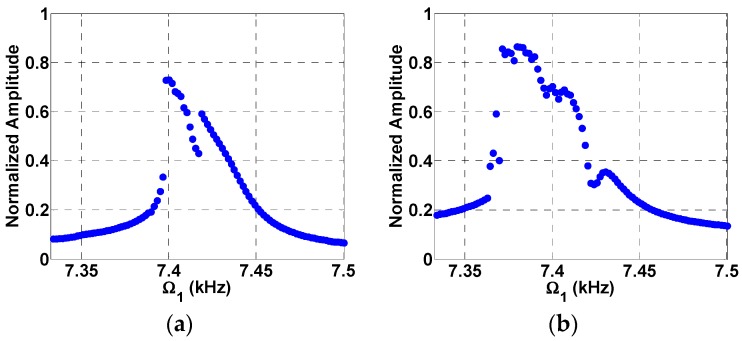
Frequency response curves near superharmonic resonances and two source excitation at $V_{\mathrm{DC}}=1\;V$, $V_{\mathrm{AC}1}=0.35\;V$ (**a**) $V_{\mathrm{AC}2}=0.15\;V$, $\Omega_{2}=7.5\;\mathrm{kHz}$; (**b**) $V_{\mathrm{AC}2}=0.25\;V$, $\Omega_{2}=7.5\;\mathrm{KHz}$.

Based on the parameters provided in Table 2, the natural frequency of the beam is found to be around $\Omega_{n}=15\;\mathrm{kHz}$, while the static pull-in voltage is found to be 5.42 V. Figure 5 shows the frequency response curve near superharmonic resonance of order two (near half the natural frequency) for two loading cases, a single AC excitation as shown in Figure 5a and a two AC excitation source as shown in Figure 5b. The normalized displacement is plotted versus frequency. Figure 5a shows the superharmonic resonance at $\Omega_{n}/2$ triggered by electrostatic nonlinearity due to a single AC source [19,20]. The maximum normalized amplitude achieved was found to be 0.52 for $V_{\mathrm{DC}}=1\;V$ and $V_{\mathrm{AC}1}=0.35\;V$. On the other hand, in Figure 5b the system was excited with two AC loads, $V_{\mathrm{AC}1}=0.35\;V$ and $V_{\mathrm{AC}2}=0.1\;V$, and with a fixed frequency of $\Omega_{2}=\Omega_{n}/2=7.5\;\mathrm{kHz}$. In addition to triggering of the superharmonic resonance by the quadratic electrostatic nonlinearity, the superharmonic resonance is triggered by another subtractive combination frequency at $\Omega_{n}-\Omega_{2}=7.5\;\mathrm{kHz}$. This means that the superharmonic resonance is now triggered by two sources; one from the electrostatic nonlinearity while the other is from frequency mixing. This lead to a higher normalized amplitude near 0.6 as shown in Figure 5b due to the new AC load compared to 0.52 in Figure 5a. The mixed response also shows two peaks corresponding to the two different sources of resonances. A further increase in the $V_{\mathrm{AC}2}$ voltage from 0.15 V in Figure 6a to in Figure 6b leads to a further increase in the normalized amplitude from 0.68 to 0.84.

**Figure 7:**
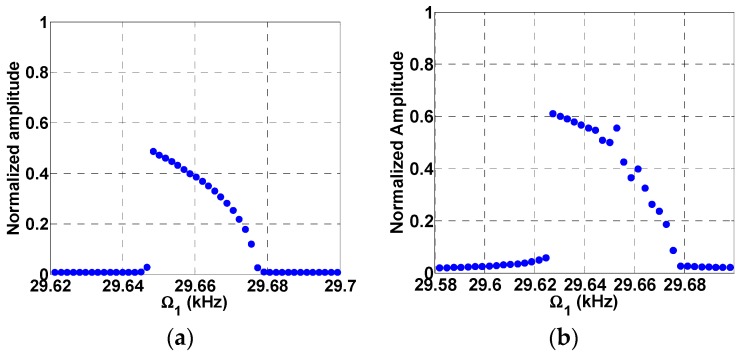
Subharmonic frequency response curve for $V_{\mathrm{DC}}=1\;V$, $V_{\mathrm{AC}1}=0.15\;V$ and (**a**) Single source excitation, $V_{\mathrm{AC}2}=0$; (**b**) two source excitation at $V_{\mathrm{AC}2}=0.01\;V$, $\Omega_{2}=15\;\mathrm{kHz}$.

**Figure 8:**
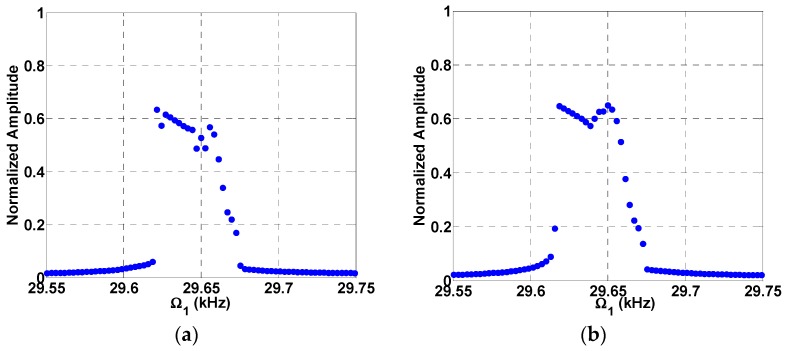
Subharmonic frequency response curve for $V_{\mathrm{DC}}=1\;V$, $V_{\mathrm{AC}1}=0.15\;V$, (**a**) $V_{\mathrm{AC}2}=0.013\;V$; (**b**) $V_{\mathrm{AC}2}=0.017\;V$, $\Omega_{2}=15\;\mathrm{kHz}$.

Next, we investigate the effect of multi-frequency excitation around sub-harmonic resonance. Figure 7a shows that the normalized tip displacement is plotted versus frequency under a single AC load. The subharmonic resonance is triggered here at $2\Omega_{n}\approx 30\;\mathrm{kHz}$ due to the electrostatic nonlinearity with a maximum normalized amplitude of 0.5. In Figure 7b, the system was excited with two AC loads, $V_{\mathrm{AC}1}=0.15\;V$ and $V_{\mathrm{AC}2}=0.01\;V$, and with a fixed frequency of $\Omega_{2}=\Omega_{n}=15\;\mathrm{kHz}$. In addition to the resonance of Figure 7a, a combination resonance frequency of $\Omega_{n}+\Omega_{2}=30\;\mathrm{kHz}$ is triggered, which leads to a higher normalized amplitude of 0.6. Further increase in the value of $V_{\mathrm{AC}2}$ leads to higher amplitudes of 0.61 and 0.62 as shown in Figure 8a,b, respectively.

Therefore, one can note from the above results the interesting effect of multi-frequency excitation in getting higher amplitude oscillation and activating the super and sub-harmonic resonances, which in turn increases the sensitivity of these resonators when used as sensors.

## 3. Case III: Response to Three Harmonic Sources

Next, we investigate the response of a cantilever beam resonator under three-source excitation. The geometrical properties of the resonator are shown in Table 3.

**Figure 9:**
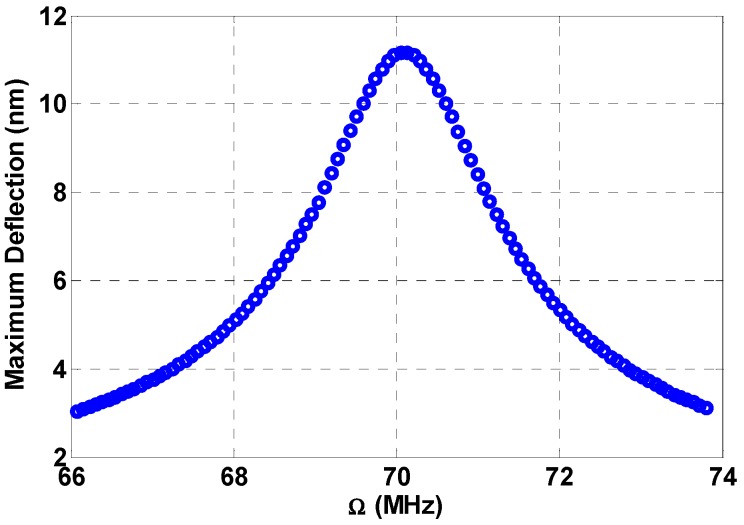
Frequency response curve near beam primary resonance at $V_{\mathrm{DC}}=5\;V$, $V_{\mathrm{AC}1}=2\;V$, $V_{\mathrm{AC}2}=0$, and c=0.1.

Figure 9 shows the frequency response curve for the beam when excited by a single source excitation near the beam natural frequency, which is equal to 70.37 MHz.

**Figure 10:**
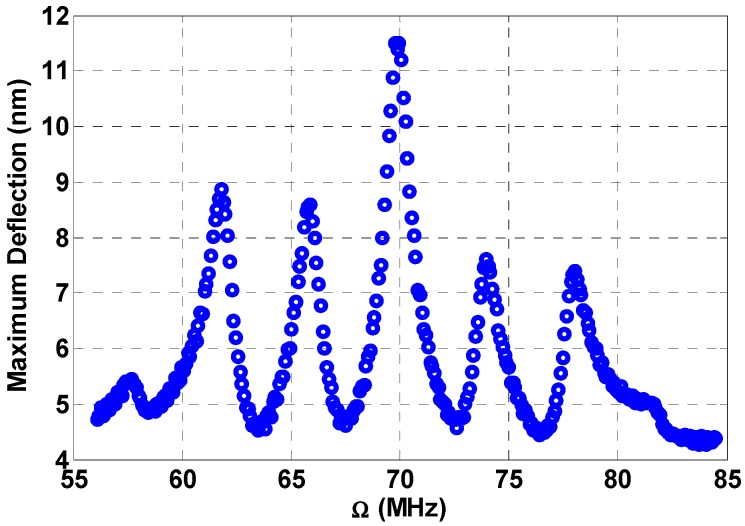
Multifrequency response near the beam primary resonance due to three-source excitation at $V_{\mathrm{DC}}=5\;V$, $V_{\mathrm{AC}1}=0.7\;V$, $V_{\mathrm{AC}2}=5\;V$, $V_{\mathrm{AC}3}=5\;V$, $\Omega_{2}=4\;\mathrm{MHz}$, $\Omega_{3}=8\;\mathrm{MHz}$, c=0.1.

Figure 10 shows the frequency response for a load of 5 V $V_{\mathrm{DC}}$, 0.7 V $V_{\mathrm{AC}1}$, 5 V $V_{\mathrm{AC}2}$, 5 V $V_{\mathrm{AC}3}$, 4 MHz $\Omega_{2}$, 8 MHz $\Omega_{3}$, and 0.1 non-dimensional damping. The maximum amplitude of 11.5 nm in the figure is due to the primary resonance, while the peaks very close to the primary resonance at 66 MHz and 74 MHz are due to the combination resonances ($\Omega_{1}+\Omega_{2}$). The peaks, which are shown farther from the primary resonance, at 62 MHz and 78 MHz, are the combination resonance created from the primary resonance and the second fixed frequency ($\Omega_{1}+\Omega_{3}$).

**Figure 11:**
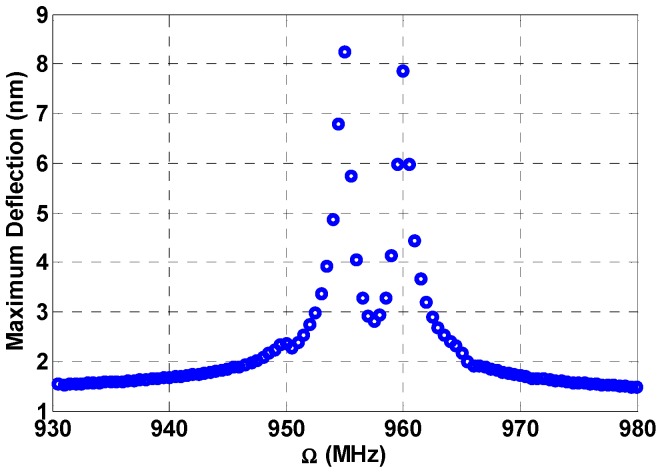
Multi-frequency excitation off-resonance: $V_{\mathrm{DC}}=3\;V$, $V_{\mathrm{AC}1}=1\;V$, $V_{\mathrm{AC}2}=6\;V$, $V_{\mathrm{AC}3}=6\;V$, $\Omega_{2}=885\;\mathrm{MHz}$, $\Omega_{3}=890\;\mathrm{MHz}$, c=0.1.

Now the multi-frequency response is demonstrated at a range away from the primary resonance range. In Figure 11, the frequency is swept starting from an initial value of 930 MHz and ending with a final value of 980 MHz, which is considered in the off-resonance range. The load is taken to be 3 V, while the fixed frequencies are at 885 MHz and 890 MHz, respectively. According to the values of the fixed frequencies, two additive combination peaks, $\Omega_{2}+\Omega_{n}=958.29\;\mathrm{MHz}$ and $\Omega_{3}+\Omega_{n}=960.37\;\mathrm{MHz}$, are created as shown in Figure 11. The bandwidth of the individual peaks is around 1 MHz. By controlling the fixed frequencies, we can control the bandwidth of these peaks and a wider bandwidth can be achieved.

**Figure 12:**
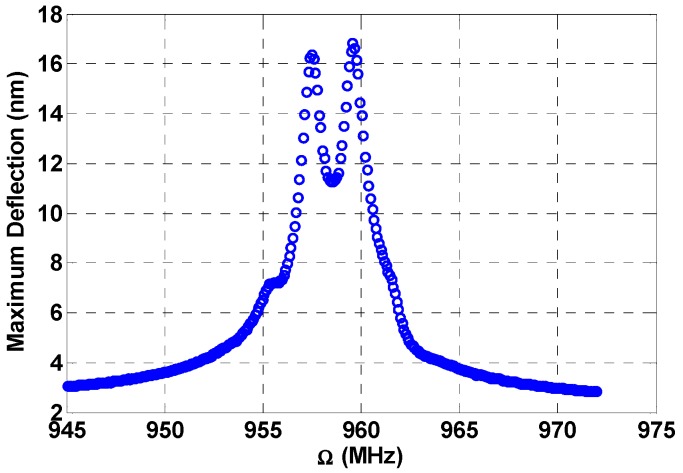
Multi-frequency excitation off-resonance: $V_{\mathrm{DC}1}=5\;V$, $V_{\mathrm{DC}2}=0\;V$, $V_{\mathrm{AC}1}=1.5\;V$, $V_{\mathrm{AC}2}=6\;V$, $V_{\mathrm{AC}3}=8.5\;V$, $\Omega_{2}=887.72\;\mathrm{MHz}$, $\Omega_{3}=887.92\;\mathrm{MHz}$, c=0.1, amplified response due to higher loads.

To amplify the response in the previous figure, we use different loads in Figure 12. The $V_{\mathrm{DC}}$ bias is set at 5 V and the $V_{\mathrm{AC}}$ voltage for the sweep is at 1.5 V, while the first and second fixed $V_{\mathrm{AC}}$ voltages are at 6 V and 8.5 V, respectively. Here we can see the individual peaks created by the additive combination resonances at $\Omega_{2}+\Omega_{n}=958.09\;\mathrm{MHz}$ and $\Omega_{3}+\Omega_{n}=960.37\;\mathrm{MHz}$. A higher response of 17 nm is achieved.

**Figure 13:**
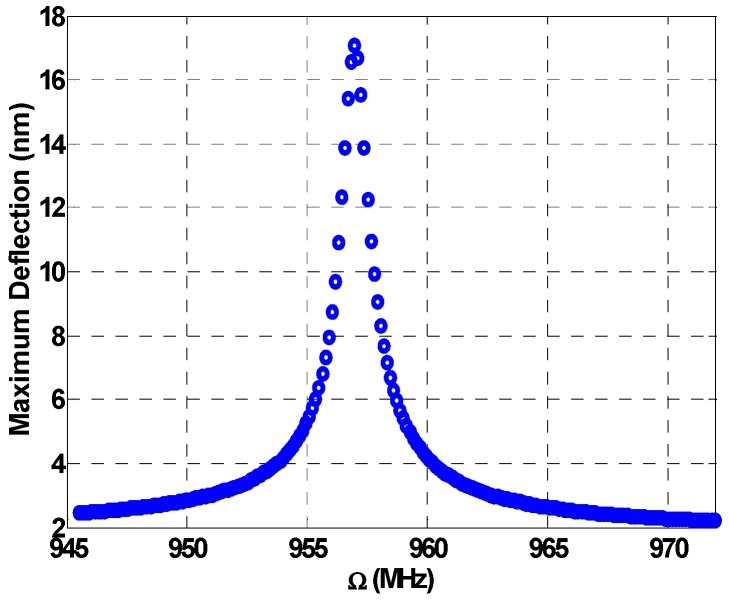
Multi-frequency excitation off-resonance: $V_{\mathrm{DC}}=5\;V$, $V_{\mathrm{AC}1}=1\;V$, $V_{\mathrm{AC}2}=6\;V$, $V_{\mathrm{AC}3}=8\;V$, $\Omega_{2}=887.72\;\mathrm{MHz}$, $\Omega_{3}=887.92\;\mathrm{MHz}$, c=0.1, the two peaks merge as one combined peak.

Figure 13 shows another result for more amplified resonance under 5 V $V_{\mathrm{DC}}$ and 8 V $V_{\mathrm{AC}3}$, while the remaining loads remain similar to the loads in the previous figure. In this figure, we choose $\Omega_{2}$ and $\Omega_{3}$ values to be very close to each other, 887.72 MHz and 887.92 MHz, respectively, to produce two additive combining peaks of $\Omega_{2}+\Omega_{n}=958.09\;\mathrm{MHz}$ and $\Omega_{3}+\Omega_{n}=958.29\;\mathrm{MHz}$. These two peaks merge as one amplified peak. We can notice a significant increase of 1.6 MHz in the bandwidth in comparison with 1 MHz for the individual peaks from Figure 11.

## 4. Experimental Case Study

**Figure 14:**
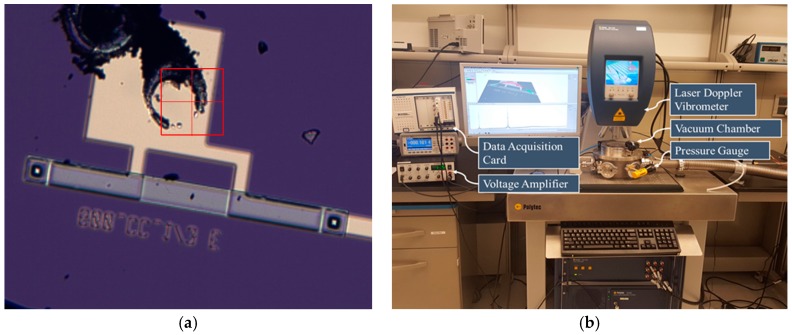
(**a**) Top view of the fabricated microbeam; (**b**) experimental setup used for testing the microelectromechanical systems (MEMS) devices.

In this section, we demonstrate experimentally the multi-frequency excitation based on a clamped-clamped microbeam of 600 µm micro length and with two-thirds electrode actuation at a pressure = 3.6 mTorr (0.4 Pa). The experimental results are validated with simulation results after we extracted the parameters in Table 4 using the parameter extraction procedure and the model reported in [16]. Figure 14a shows a top view picture of the fabricated beam. The microbeam is made of 6 µm polyimide coated with 500 nm nickel from the top and a chrome/gold/chrome layer from the bottom with thicknesses of 50/250/50 nm, which forms the upper electrode of the resonator. The lower electrode spans two thirds of the beam length and is fabricated by sputtering 50 nm chrome and 250 nm gold. The two electrodes are separated by a 2 µm air gap. The fabrication details are presented in [15,16]. The experimental setup used to test the beam is presented in Figure 14b. The setup is composed of a high frequency laser-Doppler vibrometer, a micro system analyzer (MSA-500) to measure the vibration amplitude, a data acquisition card connected with an amplifier to provide the actuation signals of a wide range of frequencies and amplitudes, and a vacuum chamber equipped with ports to pass the actuation signal and measure the pressure. Additionally, the chamber is connected to a vacuum pump. The frequency response curves are generated by taking the steady state maximum amplitude of the motion.

**Figure 15:**
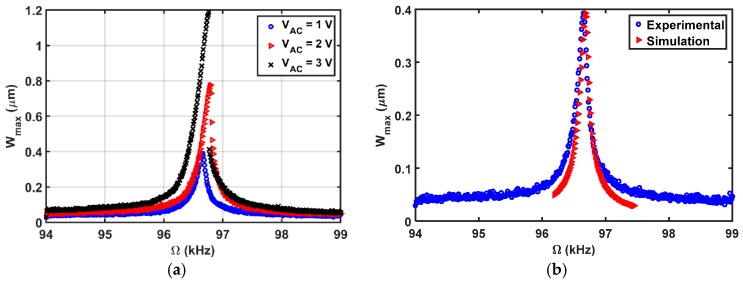
Frequency sweep results near the primary resonance of the first mode at $V_{\mathrm{DC}}=3\;V$ (**a**) different $V_{\mathrm{AC}}$ values; (**b**) $V_{\mathrm{AC}}=2\;V$.

First, the beam is tested by a DC load and a single AC source near its primary resonance, shown in Figure 15a. As the AC amplitude is increased, a hardening behavior of the beam is observed. The simulations are conducted using a multi-mode reduced order model and the Runge Kutta time integration [16].

**Figure 16:**
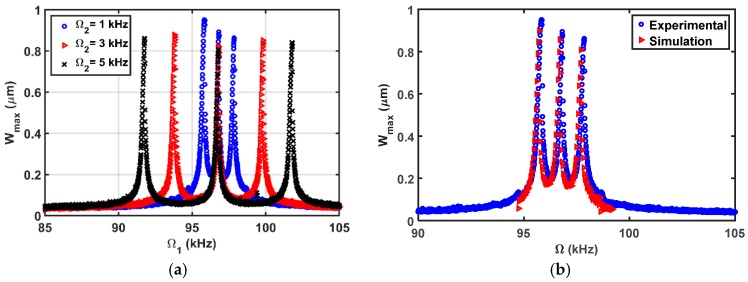
Resonances using two-source excitation near the first mode at $V_{\mathrm{DC}}=3\;V$, $V_{\mathrm{AC}1}=2\;V$, $\Omega_{1}=$ Swept, $V_{\mathrm{AC}2}=6\;V$, (**a**) $\Omega_{2}=$ as shown; (**b**) $\Omega_{2}=1\;\mathrm{kHz}$.

**Figure 17:**
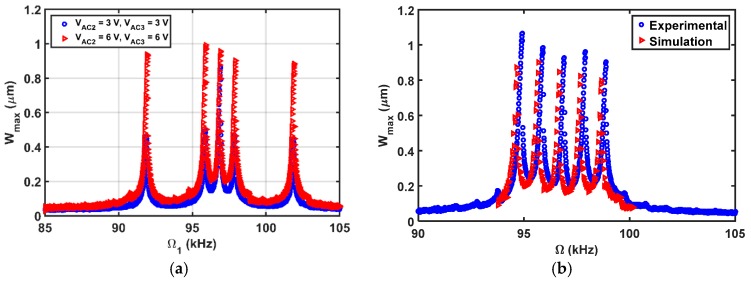
Resonances using three-source excitation near the first mode at $V_{\mathrm{DC}}=3\;V$, $V_{\mathrm{AC}1}=2\;V$, $\Omega_{1}=$ Swept, $\Omega_{2}=1\;\mathrm{kHz}$ (**a**) $V_{\mathrm{AC}2}=\mathrm{as}\;\mathrm{shown}$, $V_{\mathrm{AC}3}=\mathrm{as}\;\mathrm{shown}$, $\Omega_{3}=5\;\mathrm{kHz}$; (**b**) $V_{\mathrm{AC}2}=6\;V$, $V_{\mathrm{AC}3}=6\;V$, $\Omega_{3}=2\;\mathrm{kHz}$.

**Figure 18:**
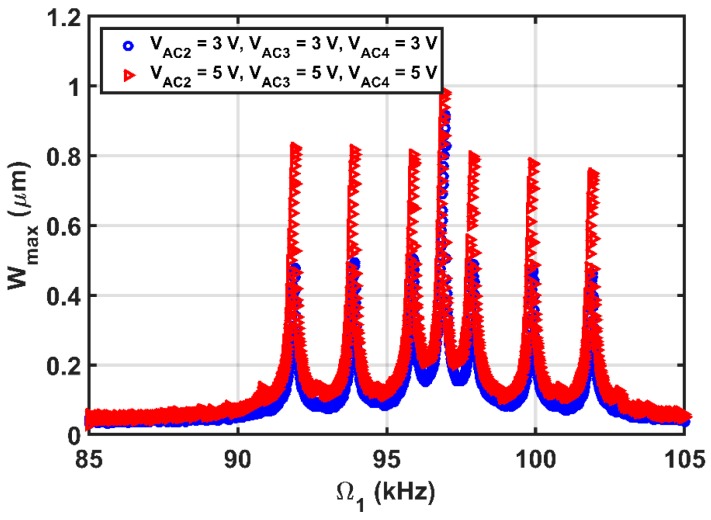
Resonances using four-source excitation near the first mode at $V_{\mathrm{DC}}=3\;V$, $V_{\mathrm{AC}1}=2\;V$, $\Omega_{1}=\mathrm{Swept}$, $V_{\mathrm{AC}2}=\mathrm{as}\;\mathrm{shown}$, $\Omega_{2}=1\;\mathrm{kHz}$, $V_{\mathrm{AC}3}=\mathrm{as}\;\mathrm{shown}$, $\Omega_{3}=3\;\mathrm{kHz}$, $V_{\mathrm{AC}4}=\mathrm{as}\;\mathrm{shown}$, $\Omega_{4}=5\;\mathrm{kHz}$.

The simulated results are in good agreement with the experimental results as shown in Figure 15b. Then, the beam is subjected to a DC load with two AC harmonic excitation loads, as shown in Figure 16, three AC harmonic loads, as shown in Figure 17, and four AC loads, as shown in Figure 18. In these figures, one frequency source is swept around the primary resonance, while the others are set at fixed values of frequencies, which are selected to be small to generate additive and subtractive resonances around the primary resonance. One can notice that for every AC source added, two near peaks around the primary resonance are generated. These experimental and simulation results confirm the attractive feature of multi-frequency excitations.

## 5. Conclusions

Modeling and simulation for a MEMS cantilever beam resonator under multi-excitation frequency have been presented. A multi-mode Reduced-Order-Model has been extracted through applying the Galerkin discretization method. Different case studies were taken into consideration. Static and dynamic analyses were carried out to explore the behavior of the resonator. From Case I, it has been found that multi-frequency excitation offers down-conversion of the RF signal from a frequency of 960 MHz to around 50 MHz. It showed the realization of the mixing and filtering of RF signals with a nominal bandwidth and good enough maximum amplitude. In Case II, mixing at secondary resonances (superharmonic and subharmonic) is demonstrated. In Case III, multi-frequency excitation using three sources has been demonstrated. Finally, an experimental case study confirming the conclusions in Case III was demonstrated based on a clamped-clamped beam. In conclusion, multi-frequency excitation has been studied in depth, revealing various exciting possibilities to utilize it for practical applications.

## Figures and Tables

**Table 1:** Geometric and physical parameters Case I.

Parameter	Value
Young’s Modulus (E)	160 Gpa
Density (ρ)	$2332\;\mathrm{Kg}/m^{3}$
Beam length (*L*)	2 μm
Beam width (b)	200 nm
Beam thickness (h)	158 nm
Gab (d)	75 nm

**Table 2:** Geometric and physical parameters for Case II.

Parameter	Value
E	82.7 Gpa
ρ	$1400\;\mathrm{Kg}/m^{3}$
$c_{\mathrm{non}}$	0.00289
*L*	500 μm
b	50 μm
*h*	3 μm
d	3 μm

**Table 3:** Geometric and physical parameters for Case III.

Parameter	Value
Young’s Modulus (E)	160 Gpa
Density (ρ)	$2332\;\mathrm{Kg}/m^{3}$
Beam length (L)	1.85 μm
Beam width (b)	200 nm
Beam thickness (h)	180 nm
Gab (d)	75 nm

**Table 4:** Parameters extracted experimentally.

Parameter	Value
Non-dimensional axial force (Nnon)	76.3
Flexural regidity (EI)	2.93 × 10^−11^ N·m^2^
Damping ratio	6 × 10^−4^
